# Numerical study of COVID-19 spatial–temporal spreading in London

**DOI:** 10.1063/5.0048472

**Published:** 2021-04-20

**Authors:** Jie Zheng, Xiaofei Wu, Fangxin Fang, Jinxi Li, Zifa Wang, Hang Xiao, Jiang Zhu, Christopher Pain, Paul Linden, Boyu Xiang

**Affiliations:** 1 Center for Excellence in Regional Atmospheric Environment, Institute of Urban Environment, Chinese Academy of Sciences, Xiamen 361021, China; 2 Applied Modelling and Computation Group, Department of Earth Science and Engineering, Imperial College London, Prince Consort Road, London SW7 2AZ, United Kingdom; 3 International Center for Climate and Environment Sciences, Institute of Atmospheric Physics, Chinese Academy of Sciences, Beijing 100029, China; 4 Department of Applied Mathematics and Theoretical Physics, University of Cambridge, Wilberforce Road, Cambridge, England CB3 0WA, United Kingdom; 5 State Key Laboratory of Atmospheric Boundary Layer Physics and Atmospheric Chemistry, Institute of Atmospheric Physics, Chinese Academy of Sciences, Beijing 100029, China; 6 Department of Atmospheric Sciences, University of Chinese Academy of Sciences, Beijing, China; 7 Wilson's School, Mollison Drive, Wallington, Surrey SM6 9JW, United Kingdom

## Abstract

A recent study reported that an aerosolized virus (COVID-19) can survive in the air for a few hours. It is highly possible that people get infected with the disease by breathing and contact with items contaminated by the aerosolized virus. However, the aerosolized virus transmission and trajectories in various meteorological environments remain unclear. This paper has investigated the movement of aerosolized viruses from a high concentration source across a dense urban area. The case study looks at the highly air polluted areas of London: University College Hospital (UCH) and King's Cross and St Pancras International Station (KCSPI). We explored the spread and decay of COVID-19 released from the hospital and railway stations with the prescribed meteorological conditions. The study has three key findings: the primary result is that the concentration of viruses decreases rapidly by a factor of 2–3 near the sources although the virus may travel from meters up to hundreds of meters from the source location for certain meteorological conditions. The secondary finding shows viruses released into the atmosphere from entry and exit points at KCSPI remain trapped within a small radial distance of < 50 m. This strengthens the case for the use of face coverings to reduce the infection rate. The final finding shows that there are different levels of risk at various door locations for UCH; depending on which door is used there can be a higher concentration of COVID-19. Although our results are based on London, since the fundamental knowledge processes are the same, our study can be further extended to other locations (especially the highly air polluted areas) in the world.

## I. INTRODUCTION

The earliest confirmed case of COVID-19 was in December 2019, and the disease has since become a global pandemic with over 125 711 889 confirmed cases and 2 760 406 deaths worldwide as of March 25, 2021 (https://www.worldometers.info/coronavirus/).

Existing studies showed that the virus is mostly spread through breathing, coughing and sneezing (Chakraborty and Maity, 2020; Howard *et al.,* 2020; Talaat *et al.,* 2021; Zhou and Zou, 2021), and direct contact with unsterilized abiotic surfaces such as plastics and stainless steel (where the virus can remain infectious for 28 days, https://www.bbc.co.uk/news/health-54500673). Social distancing in the range 1–2.5 m has been recommended to mitigate this situation (Blocken *et al.,* 2020; Wei and Li, 2015). However, there is evidence that the virus can remain in the air for more prolonged periods of time (remaining infectious for over 3 h after expulsion, van Doremalen *et al.*, 2020; Setti *et al.*, 2020), traveling further contained within aerosols suspended in the air (Li *et al.*, 2020; Narayanan and Yang, 2021). Studies have found that similar particles containing viral matter can travel up to 10 m, suggesting that the recommended social distancing may be insufficient (Morawska and Cao, 2020). A higher rate of mortality is also linked with an increased concentration of particulate matter (PM, Wu *et al.,* 2020), and the number of COVID-19 cases has been linked with the level of PM in Italy and France (Kowalski and Konior, 2020).

Prior research on the effect of aerosolized viruses in indoor transmitting the virus suggests measures like better ventilation to keep the aerosols outside of buildings, keeping those within safe (Morawska and Cao, 2020; Foster and Kinzel, 2021) but few have considered the effect of aerosols on outdoor transmission over longer distances, partly because there are no simple methods to collect data and therefore a lack of data to analyze (Carducci *et al.,* 2020).

The aim of this computational study is to tackle the aforementioned issues with the computational fluid dynamics (CFD) model Fluidity, developed by Imperial College. Fluidity is an open source large eddy simulation (LES) model with an advanced adaptive mesh capability (https://github.com/fluidityproject). This study investigates the effect of the exponential decay of the virus and the complexity of the spreading phenomenon: how long can the virus spread for given certain meteorological conditions? We will explore these in two different locations in London: University College Hospital (UCH); King's Cross Station and St Pancras International (KCSPI). This study will explore the spread of this virus from hospitals and railway stations, and the impact of meteorological conditions on this virus. Our aim is to explore the impact of the aerosol transport of the virus under specific meteorological conditions and highlight the importance of treating the pandemic seriously. Although our study is based on London, it can be extended to other locations (especially the highly air polluted areas) in the world since all the fundamental knowledge principles are the same.

## II. METHODOLOGY

### A. Governing equations in virus spreading simulations

The three-dimensional (3D) Navier–Stokes equations and generic atmospheric transport (advection-diffusion) equation are utilized for COVID-19 spreading simulations. The 3D Navier–Stokes equations are written as

$$\frac{\partial u}{\partial t}+u\cdot \nabla u=-\frac{1}{\rho }\nabla p+S_{u}+\mu \nabla^{2}u,$$ (1)

where $u={\left(u,v,w\right)}^{T}$ is the velocity vector, *t* is the time, $\nabla =\frac{\partial }{\partial x}i+\frac{\partial }{\partial y}j+\frac{\partial }{\partial z}k$, *ρ* is the (assumed uniform) density of the atmosphere, $S_{u}$ represents the source, absorption, or the drag forcing term of velocity (e.g., there is an absorption term when the flow passes the trees), and μ represents the dynamic viscosity.

The COVID-19 virus transport equation is

$$\frac{\partial C}{\partial t}+\nabla \cdot \left(uC\right)-\nabla \cdot \left(\bar{\kappa }\nabla C\right)=S+D,$$ (2)

where *C* is the mass concentration of the virus, $\bar{\kappa }$ is the tensor of turbulent diffusivity, *S* represents the source term of virus, and *D* is the decay term of virus. In this study, the aerosol physical interaction between particles of different diameters is not considered. The emitted virus concentration around the source locations is positively correlated with the around aerosol concentration. This article investigates the potential pathways routes for COVID-19-saturated aerosol flow from local sources.

The virus exponential decay formulation (van Doremalen *et al.,* 2020) is given as follows:

$$C=C_{0}\cdot e^{-\lambda t},$$ (3)

where $C_{0}$ is the initial virus concentration and *λ* denotes the decay rate. The decay term in Eq. (2) can thus be expressed as D=−λC. Studies have shown that the decay of airborne viruses is sensitive to the metrological conditions (wind velocity, ambient humidity, and temperature Yang and Marr, 2012; Schuit *et al.,* 2020; Chan *et al.,* 2011; Dbouk and Drikakis 2020; Dabisch *et al.*, 2021). In this work, to ensure stability and suppress spurious oscillations, the control volume (CV) method is used for resolving the virus concentration. To avoid spurious oscillations, the CV–TVD (control volume—total variation diminishing) limiter is used to make the solutions total variation diminishing. For control volume discretization, an explicit scheme is simple but strictly limited by the CFL number, which can be restrictive on adaptive meshes as the minimum mesh size can be very small. We adopt a new time stepping scheme based on traditional Crank–Nicolson scheme because of its robustness, unconditional stability, and second-order accurate in time (Zheng *et al.,* 2015, Pavlidis *et al.,* 2015).

### B. Meteorological boundary conditions

The synthetic eddy method (Pavlidis *et al.,* 2010) is used to setup the inlet boundary condition

$$U_{in}\left(x,t\right)={\bar{U}}_{in}\left(x\right)+{u^{\prime }}_{in}\left(x,t\right),V_{in}\left(x,t\right)=W_{in}\left(x,t\right)=0,$$ (4)

where x=(x,y,z) are the spatial coordinates, *t* is the time, $U_{in}\left(x,t\right),V_{in}\left(x,t\right),W_{in}\left(x,t\right)$ are the velocity components at the inlet along the *x, y, z* directions, respectively; the mean velocity ${\bar{U}}_{in}\left(x\right)$ is the function of the vertical *z* coordinate obeying the standard log-law over roughness height *z_0_*

$${\bar{U}}_{in}\left(z\right)=\{\begin{array}{cc} 0, & z<z_{0}\\ \frac{u_{*}}{\kappa_{\alpha }}\text{ln}\;\left(\frac{z}{z_{0}}\right), & z\geq z_{0} \end{array},$$ (5)

where $u_{*}$ is the friction velocity, $\kappa_{\alpha }$ is von Karman constant, and ${u^{\prime }}_{in}\left(x,t\right)$ is the fluctuating velocity. The calculation of the fluctuating component requires the turbulence length-scale, Reynolds stresses, as well as a number of coherent structures (known as turbulent spots) (for details, see Aristodemou *et al.,* 2018).

### C. A dynamically adaptive mesh model for computational fluid dynamics (CFD) and virus spreading simulations

Fluidity is a computational fluid dynamics code capable of numerically solving the Navier–Stokes Eq. (1) with the large eddy simulation (LES) and accompanying field equations shown in Eq. (2) on unstructured meshes (for details, see AMCG, 2014; Zheng *et al.*, 2015, 2020). The key feature of Fluidity is the use of anisotropic adaptive mesh techniques. The mesh is adapted to optimally resolve the multi-scale flow dynamics in full 3D as the flow evolves in space and time.

Once viruses are emitted into the air, the dynamic and transmission processes involve a wide range of spatial scales. An artificial dilution of viruses may lead to a shorter lifetime in existing fixed grid models if the resolution of grids is not high enough. It has been proved (Zheng *et al.,* 2015, 2020) that mesh adaptivity is the most efficient and effective approach for resolving multi-scale dynamic processes. The mesh is adapted with respect to the dynamic flow features and virus concentrations in time and space. Using the adaptive mesh, the detailed flow dynamics and the temporal and spatial evolution of COVID-19 viruses during the spreading process can be captured, especially the local turbulent flows around buildings.

## III. CASE STUDY

### A. Modeling setup

**FIG. 1.**
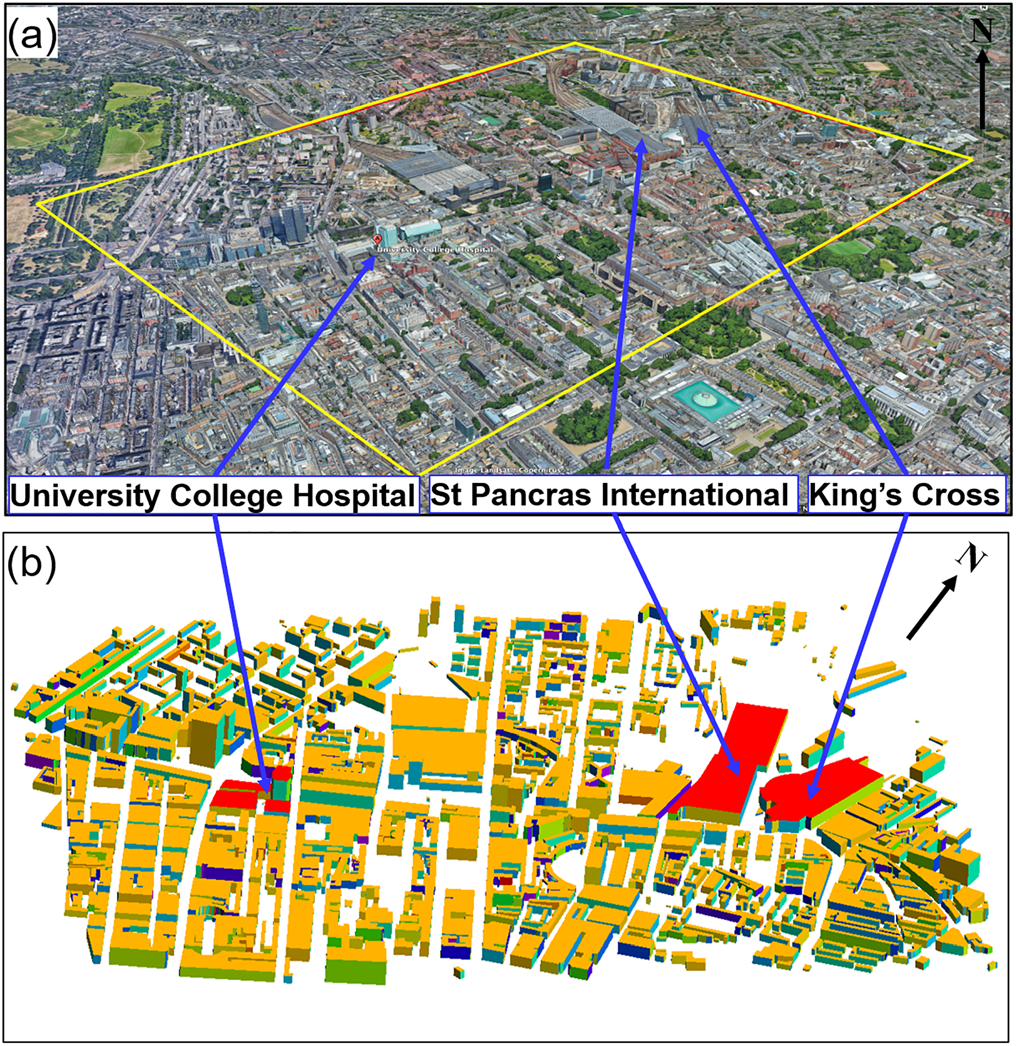
Study location and geometry in the center of London shown in (a) Google map and (b) model visualization.

**FIG. 2.**
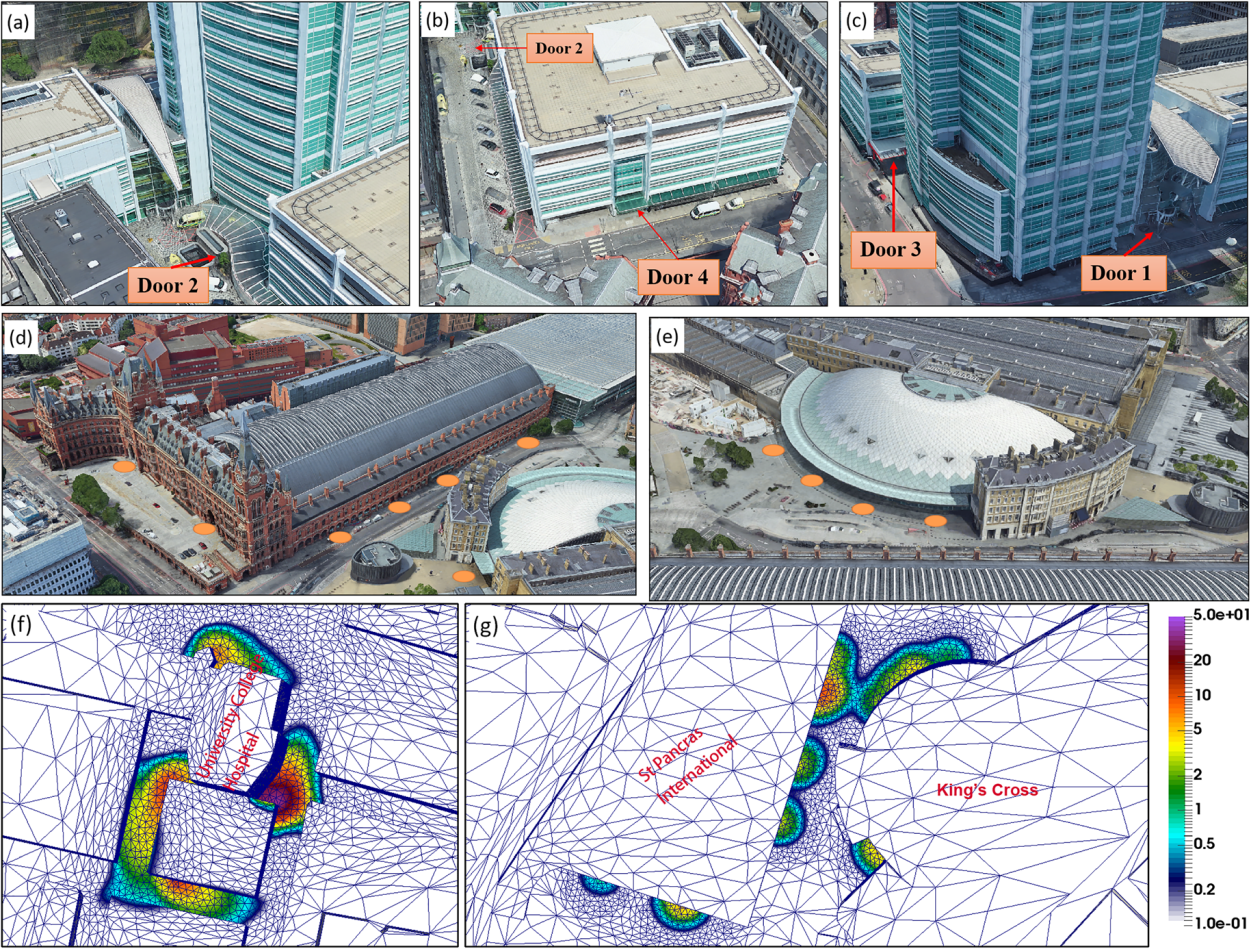
Release location of viruses in (a)–(c) Doors 1–4 in UCH, (d) the St Pancras International, and (e) King's Cross stations, as well as the corresponding initial adaptive meshes and virus concentration (unit: copies/m^3^) around the source locations (f) UCH and (g) railway stations.

• **Study area**: University College Hospital (UCH) and King's Cross and St Pancras International Station (KCSPI) on the Euston road in the center of London are close to significant traffic along Euston road, resulting in high pollution levels around that area. The computational area is a rectangular 2 × 1.5 km^2^ domain centered on UCH and 1000 m high to capture the dynamic part of the atmospheric boundary layer. As shown in Fig. 1, the computational domain includes many buildings of different heights and configurations.
• **Wind field:** In March, the wind direction is mostly from the southwest and a typical mean wind speed of approximately 5.23 m/s at 10 m above the ground (see https://weatherspark.com/m/45061/3/Average-Weather-in-March-in-City-of-London-United-Kingdom).
  **COVID-19 virus lifetime**: Given the range of the lifetime is from 1 to 4 h for COVID-19(van Doremalen *et al.*, 2020), the exponential lifetime of the virus is assumed to be 3 h here. In the outdoor simulations, the virus spread rapidly and achieve a steady state status within 10 min for the given computational domain size (2 × 1.5 km^2^). Within such a short period (10 min), the effect of the decay rate can be ignored. Compared to the role of the wind speed on the transmission and diffusion process of the virus concentration, the effect of the decay rate is relatively small in outdoor simulations.
• **Source locations and concentrations**: The locations of virus sources at UCH are chosen at the entries in Doors 1–4, shown in Figs. 2(a)–2(c). In the work of Liu *et al.*, 2020, they reported that the concentration of COVID-19 in different locations in Wuhan city is mostly less than 50 copies/m^3^ (see Table 1 in Liu *et al.*, 2020). In this study, the range of the virus concentration is set to 3–50 copies/m^3^ in the source locations [see Figs. 2(e) and 2(f)]. The residue of viable viruses at the source locations is associated with the meteorological conditions (Frontera *et al.*, 2020, Liu *et al.*, 2020, Şahin, 2020; Wang *et al.*, 2020; Briz-Redón and Serrano-Aroca, 2020).
  **Adaptive mesh resolutions**: The use of dynamically adaptive meshes optimizes the computational effort to resolve the flow dynamic and virus transport processes over a wide range of spatial scales. In this study, the mesh is dynamically adapted with respect to both the wind velocity field and virus concentration. The a priori error measure for adapting the mesh is 0.3 m/s for velocity solutions and the relative error measure is 0.01 for virus concentration. The minimum (maximum) mesh size is set to be 3 m (100 m). The maximum number of nodes is set to be 600 000, which is large enough to ensure the a priori error to be achieved. To avoid spurious dilution of virus emission, high-resolution meshes are located around the sources [see Figs. 2(f) and 2(g)].
• **Boundary and initial conditions**: At the inlet boundary, the velocity profile is given by Eq. (4). Open boundary conditions (stress-free boundary conditions) are specified at the outlet. No-slip boundary conditions are provided in the top, bottom and side boundaries. The CFD simulation starts from the “static” state.

### B. Results and discussion

In this discussion, we will focus on (1) the spatial distribution of viruses released from UCH and KCSPI for given wind field; (2) the release locations of viruses in hospital; and (3) the distance of virus transmission.

**FIG. 3.**
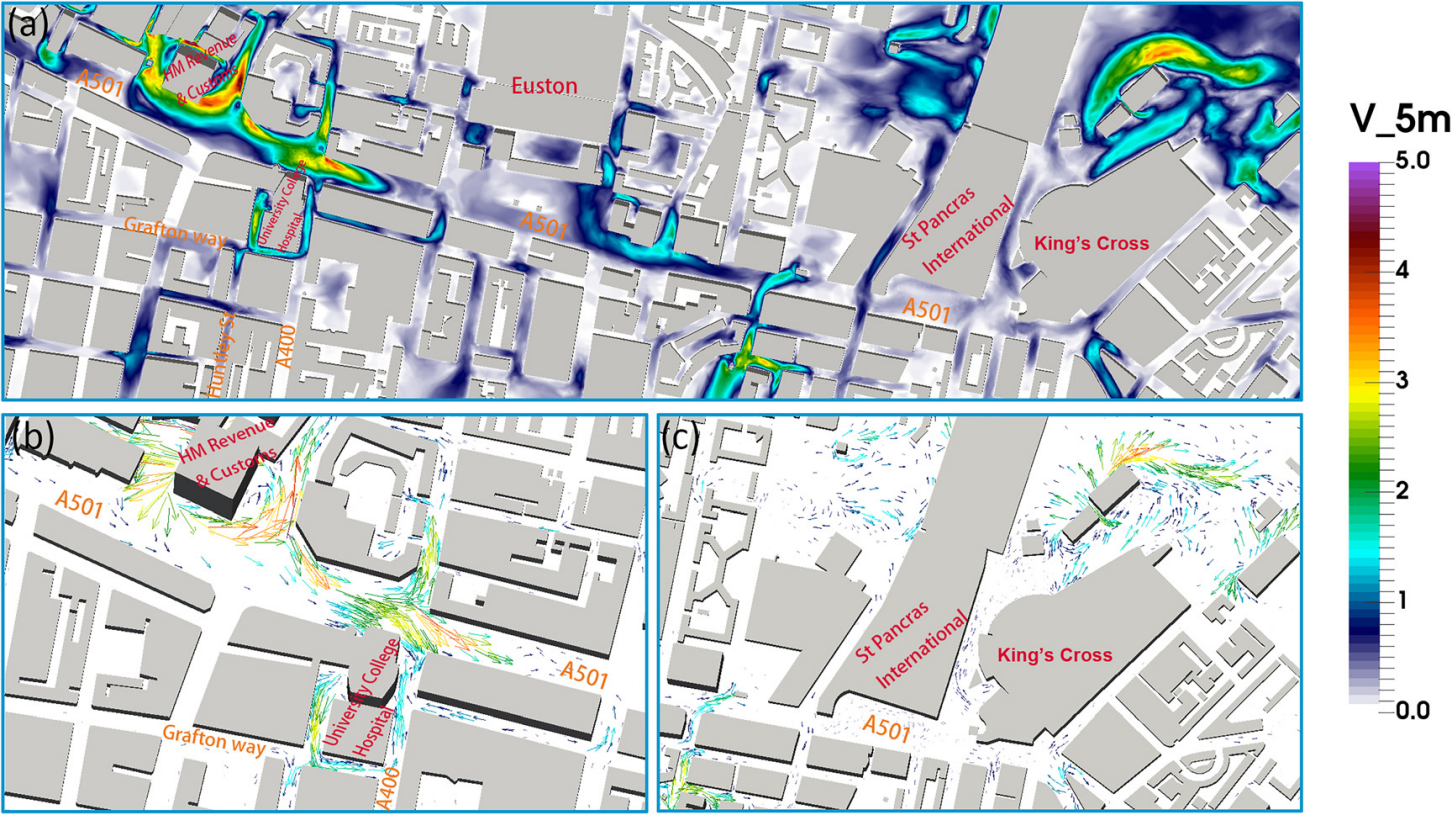
Top panel: (a) Contour map of the mean velocity field (unit: m/s) at the height of 5 m in the whole computational domain, Bottom panel: Zoom of the mean velocity field at the height of 5 m around (b) UCH, and (c) rail stations.

**Wind field and spatial distribution of viruses**: The wind velocity solution from the CFD simulations is shown in Fig. 3. We can observe how turbulent flows are developed around higher buildings—e.g., at the HM Revenue & Customs in Fig. 3 (at the intersection between A501 and A400), the wind speed can reach 4 m/s, also reaching 2.5 m/s around the UCH building (both measurements taken at a height of 2 m). If the virus is emitted from UCH, it can build up around the corner between Euston road and North Gower street. Euston road has high levels of congestion, leading to high pollution levels (over 22 *μ*g/m^3^) (see the https://www.londonair.org.uk), and the aerosol present there could transmit the disease. The increased wind speed can carry the aerosolized virus along East Euston road and North Gower street, reaching Euston Square Gardens up to KCSPI. This demonstrates how the viruses emitted from UCH can infect neighboring communities—the area around University College London (UCL)—and the commuters passing through Euston road and Gower street, to list a few.

**FIG. 4.**
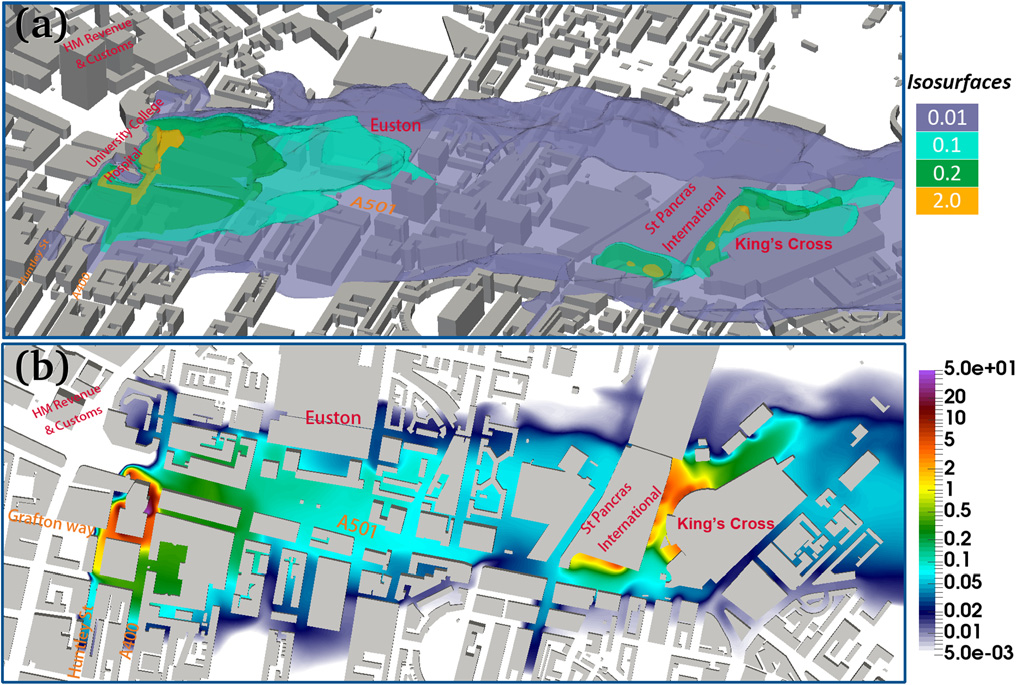
(a) 3D iso-surface of the virus concentration (unit: copies/m^3^) of viruses released from UCH, the St Pancras International, and King's Cross stations; (b) contour map of viruses at the height of 2 m, where the color bar represents the value of the virus concentration (unit: copies/m^3^).

**FIG. 5.**
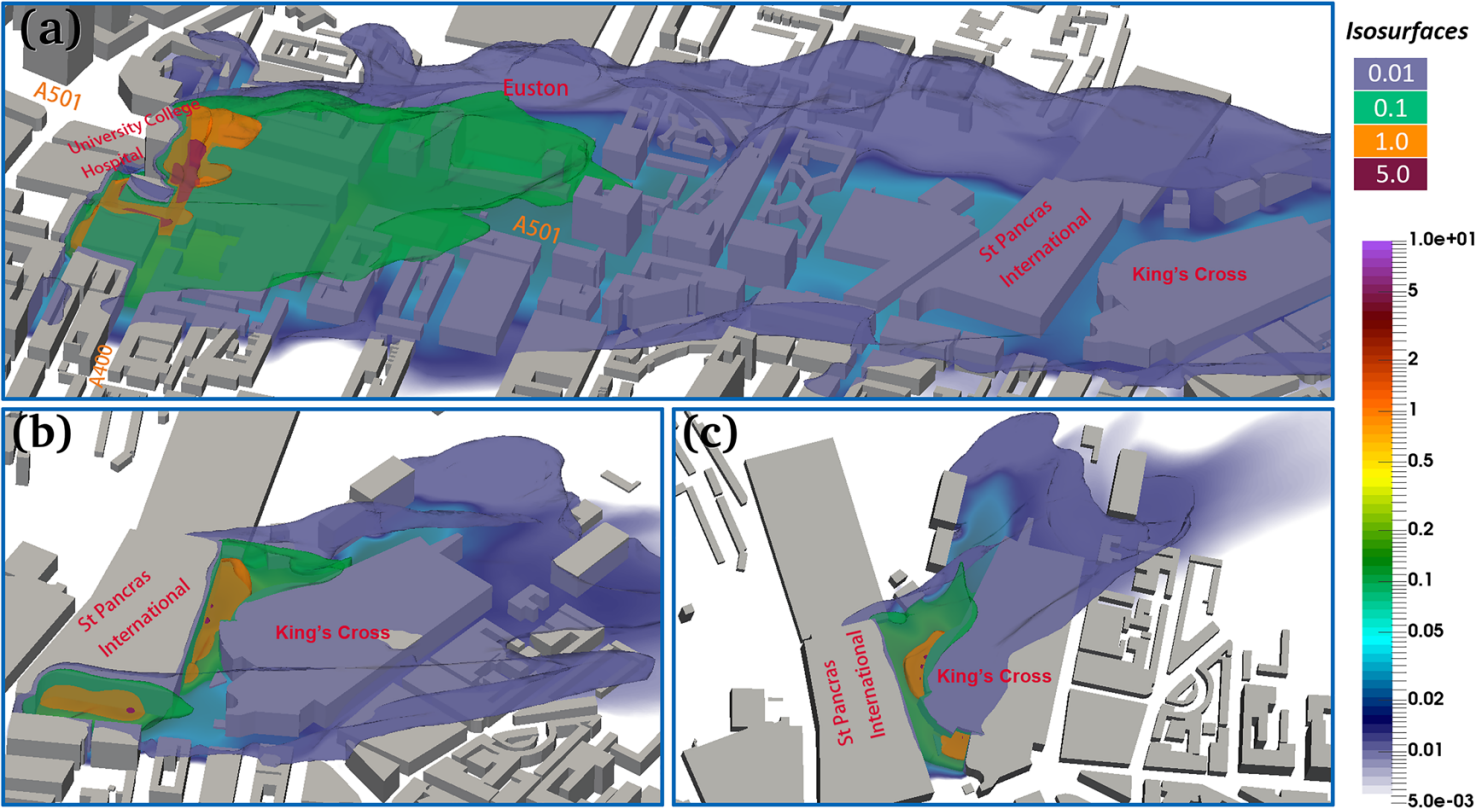
3D iso-surface of the virus concentration (copies/m^3^), where the viruses are released from (a) UCH, (b) the St Pancras International, and (c) King's Cross station where the color bar represents the value of the virus concentration (unit: copies/m^3^).

**FIG. 6.**
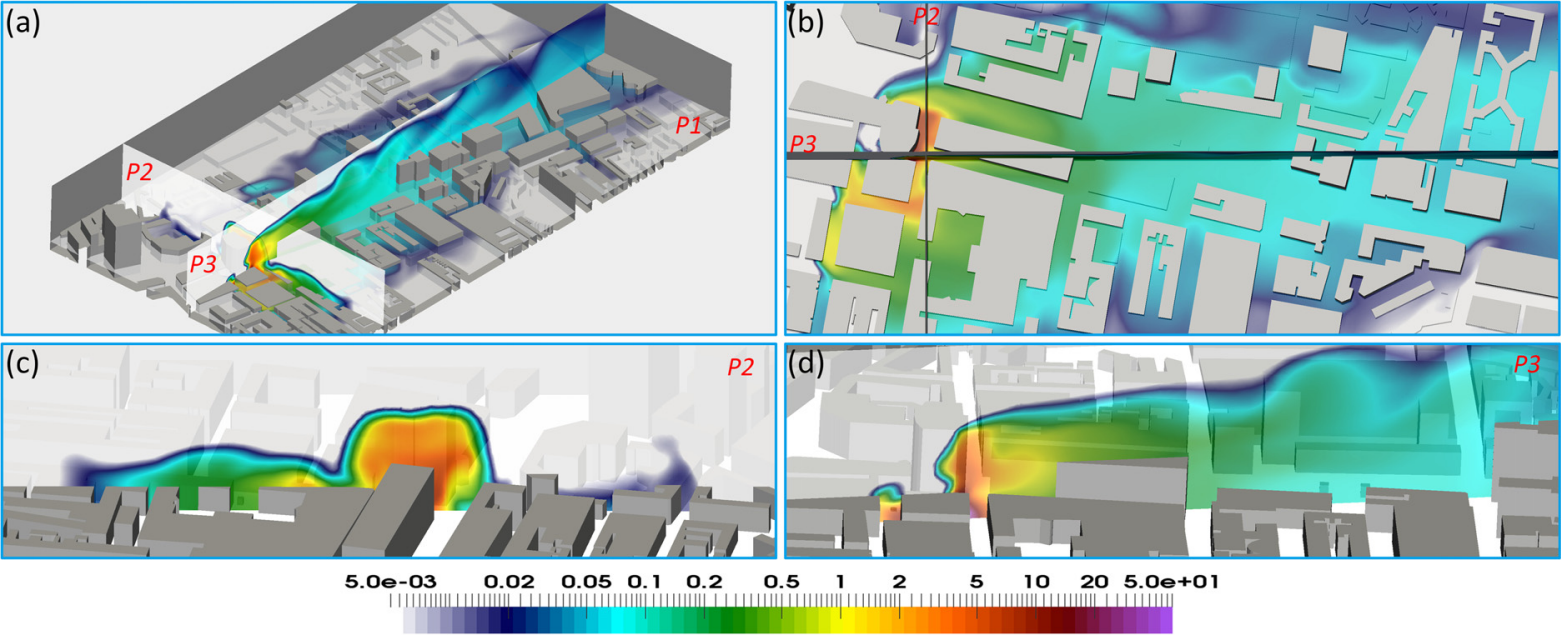
Contour map (unit: copies/m^3^) of viruses (a) overview at vertical sections. P2 and P3 over the whole domain; (b) the horizontal section at the height of 25 m, (c) the vertical section P2, and (d) vertical section P3.

**FIG. 7.**
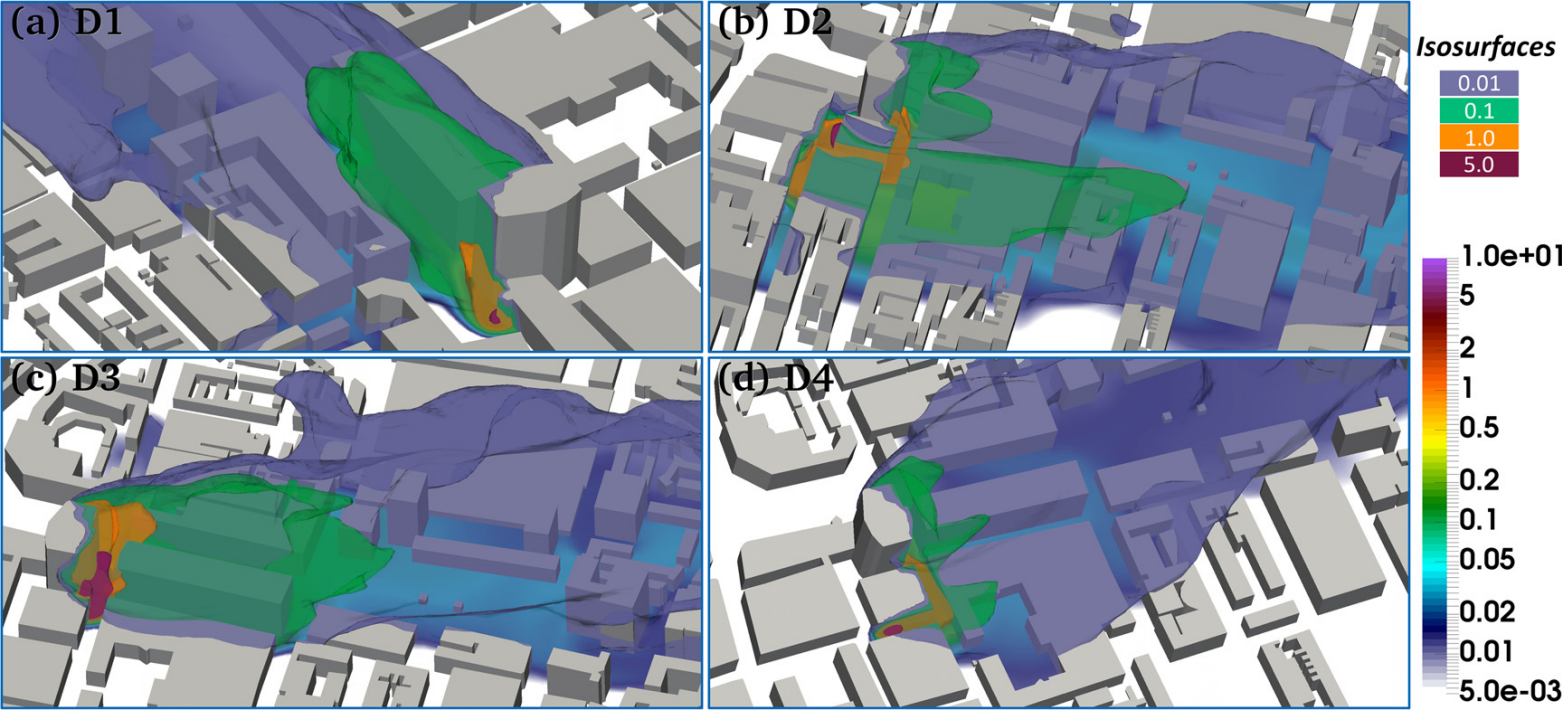
3D concentration iso-surface of viruses (copies/m^3^) released from UCH at (a) Door 1, (b) Door 2, (c) Door 3, and (d) Door 4. The color bar represents the iso-surface value of the virus concentration. The color bar represents the value of the virus concentration.

**Spatial distribution of viruses**: We have plotted the virus contour maps in Figs. 4–7. From these, we can see that the concentration of viruses decreases rapidly by a factor of 2–3 near the sources. Relatively higher concentrations of the virus (0.3–10 copies/m^3^) can be found around UCH, Gower road, A400, Gower P1, and all the way up to Euston Square Gardens. Further downwind past the Euston Square Gardens—places like the British library, St Pancras International, and King's Cross stations—the concentration is much lower, in the range of 0.1–0.2 copies/m^3^, roughly 0.1% of the concentration found in the immediate vicinity of UCH. From the contour maps, we can observe that the majority of the viruses remain trapped within a short radial distance of under 50 m along St. Pancras Road, past that, the concentration rapidly diluted to under 0.1 copies/m^3^. Concentrations around 0.1 copies/m^3^ are found within 250 m away from UCH. This can be attributed to the weaker wind field of around 0.5 m/s around the railway stations compared to the stronger wind field near UCH at 0.5–3 m/s.

Source location of viruses: We have experimented with test cases that have many different release locations (Doors 1–4, see Fig. 2) at UCH with the spatial distribution of viruses released from the doors shown in Fig. 7. It should be noted that the infectious area (see the green area representing the iso-surface of 0.1 copies/m^3^) of the virus emitted at Door 2 is larger than those of other doors. The virus released from Door 1 is directly transported to Euston road while those released from Door 2 are blown down Grafton way, splits between going down Huntley street and staying in Grafton way, some of the latter merges with the viruses from Door 4 and with Door 3's at A400, from which it moves continuously to Euston road. The infectious area of the virus emitted from Doors 1 and 2 mostly focuses on Wolfson Institute on Grafton way, UCL along A400, while the virus emitted from Doors 1 and 3 mainly infects the commuters passing through Euston road and Euston square station to list a few.

## IV. CONCLUSIONS

Our findings suggest that the aerosolized virus particles can be transmitted a long distance (hundreds of meters) due to the fully developed turbulent flows around the source locations. For example, around the UCH, there is a strong wind field (∼2.5 m/s) at the height of 5 m and viruses with the concentration of >0.2 copies/m^3^ can be found within 60–500 m away from UCH for given meteorological conditions (e.g., wind field). We also notice that the infectious area from the virus released from Door 2 in UCH is larger than the other doors. This suggests that Door 2 in UCH is not a good location for A & E; the entry location in hospital must be chosen carefully. Our study found that the majority of the viruses released from the St Pancras International and King's Cross stations remain trapped within a short radical distance of less than 50 m and will not affect the people living nearby. In summary, it is suggested that a face cover is needed for personal protection if people travel to public dense places (hospital, train stations, etc.). Finally, the impact of urban green (tree, for example) environment on reducing the virus spreading will be further investigated in our future work.

## Data Availability

The data that support the findings of this study are available within the article. For geometry data in London, please see https://www.emu-analytics.com/products/datapacks.php.
